# A social‐technological epistemology of clinical decision‐making as mediated by imaging

**DOI:** 10.1111/jep.12637

**Published:** 2016-10-03

**Authors:** Sophie van Baalen, Annamaria Carusi, Ian Sabroe, David G. Kiely

**Affiliations:** ^1^ Department of Philosophy University of Twente Enschede The Netherlands; ^2^ Medical School University of Sheffield Sheffield UK; ^3^ Department of Infection Immunity & Cardiovascular Disease University of Sheffield Sheffield UK; ^4^ University of Sheffield Sheffield UK

**Keywords:** clinical decision‐making, distributed knowing, epistemological responsibility, medical imaging, pulmonary hypertension

## Abstract

In recent years there has been growing attention to the epistemology of clinical decision‐making, but most studies have taken the individual physicians as the central object of analysis. In this paper we argue that knowing in current medical practice has an inherently social character and that imaging plays a mediating role in these practices. We have analyzed clinical decision‐making within a medical expert team involved in diagnosis and treatment of patients with pulmonary hypertension (PH), a rare disease requiring multidisciplinary team involvement in diagnosis and management. Within our field study, we conducted observations, interviews, video tasks, and a panel discussion. Decision‐making in the PH clinic involves combining evidence from heterogeneous sources into a cohesive framing of a patient, in which interpretations of the different sources can be made consistent with each other. Because pieces of evidence are generated by people with different expertise and interpretation and adjustments take place in interaction between different experts, we argue that this process is socially distributed. Multidisciplinary team meetings are an important place where information is shared, discussed, interpreted, and adjusted, allowing for a collective way of seeing and a shared language to be developed. We demonstrate this with an example of image processing in the PH service, an instance in which knowledge is distributed over multiple people who play a crucial role in generating an evaluation of right heart function. Finally, we argue that images fulfill a mediating role in distributed knowing in 3 ways: first, as enablers or tools in acquiring information; second, as communication facilitators; and third, as pervasively framing the epistemic domain. With this study of clinical decision‐making in diagnosis and treatment of PH, we have shown that clinical decision‐making is highly social and mediated by technologies. The epistemology of clinical decision‐making needs to take social and technological mediation into account.

## 1 INTRODUCTION

In recent years there has been a growing attention to the epistemology of clinical decision‐making.^1^, ^2^, ^3^, ^4^, ^5^, ^6^, ^7^, ^8^, ^9^ In this paper we argue that knowing in current medical practice has an inherently social character and that technologies such as imaging play a crucial and mediating role in these practices. The epistemology of clinical decision‐making needs to take the social and technological mediation of clinical decision‐making into account.

### 1.1 Teamwork in clinical practice

That teamwork is a crucial aspect in medical practice has been recognised in sociological studies of medical practice. For example, several studies^10^, ^11^, ^12^ draw attention to teamwork in the operating theatre, while others describe teamwork in clinical decision‐making. Cicourel^13^ characterises the clinical diagnostic process as socially distributed cognition, which “refers to the fact that participants in collaborative work relationships are likely to vary in knowledge they possess.” Within this process, “physicians typically assess the adequacy of medical information on the basis of the perceived credibility of the source, whether the source is the patient or another physician” (p. 222). Through discourse, physicians assess the area and level of expertise of their coworkers and the reliability of their patient's account of his or her illness to be able to evaluate the robustness of the information provided.

Maseide^14^ also characterise medical decision‐making as socially distributed cognition: “A ward conference, with a number of individual members with different qualifications, functions, responsibilities, skills and experiences, has knowledge and memory structures, procedures of reasoning and practical qualifications that are socially distributed and differ from the cognitive capabilities of the individual participants.” He identifies 4 forms of evidence that “influence and regulate the judgments and decisions of medical practitioners” (p. 44): scientific evidence, evidence from personal experience, evidence as medical representation artifacts, such as images and pathology results, and “practical evidence,” which is, according to Maseide, closely integrated with forms 2 and 3. This last form of evidence is cooperatively and collaboratively constructed: “practical medical evidence is generated, developed and made useful locally by medical practitioners.” (p. 44) There are several seminal studies of the social aspects of decision‐making in the tradition of distributed cognition. Cohen et al^15^, for example, considers decision‐making in the context of a psychiatric ward, drawing upon a theoretical framework initially developed by Hutchins.^16^ This framework considers cognition to be distributed across individuals and artifacts, combining internal representations (in the minds of individuals) and external representations (in physical media, such as shared whiteboards). The account proposed here covers similar terrain, in that it stresses the social—or distributed—nature of knowledge processes, and the role of media, such as images. However, as will be discussed later, there are important differences between distributed cognition theory and our own.

This paper's main concern is with epistemology, that is, it aims to shed light on the epistemology of clinical decision‐making and also to contribute to the development of philosophical epistemologies able to cope with these kinds of contexts. The paper argues that the social nature of clinical decision‐making is an ineliminable aspect of its epistemology. By this we mean that an individualist epistemology, based on a traditional analysis of knowledge in terms of individual knower, is not adequate as a basis for an account of knowledge in clinical contexts and that sociability is a necessary aspect of the epistemology of clinical decision‐making. The social character of knowledge in other spheres has been recognised by many philosophers, an early example in the turn to social knowing in scientific contexts being Hardwig^17^ who writes: “Knowing, then, is often not a privileged psychological state. If it is a privileged state at all, it is a privileged social state. So, we need an epistemological analysis of the social structure that makes the members of some teams knowers while the members of others are not.” The social structures of knowledge are increasingly acknowledged in philosophical studies of scientific practice,^17^, ^18^, ^19^, ^20^, ^21^ but this has not received the same degree of attention in philosophical accounts of clinical decision‐making. For example, Montgomery's^3^ detailed analysis of how physicians deal with uncertainty and incomplete information in clinical decision‐making focuses on the individual doctor in clinical‐patient interactions. Montgomery describes medical case conferences in one of her chapters, foregrounding the establishment of authority and hierarchy, as an aspect of the sociability of knowledge practices. Cunningham^22^ shows the extent to which the sociability of clinical decision‐making is increasingly acknowledged as a challenge in philosophy of clinical decision‐making. He uses a distributed cognition theoretical framework as the core of his normative account of clinical decision‐making. While overlapping in our concerns, the socio‐technological epistemology we propose differs in its theoretical orientation, as discussed in the concluding section of the paper. Finally, we note that the position we advocate does not imply a “collective knower” over and above group members, but we will not enter into this debate here. Rather, our interest is in showing that the epistemology of decisions regarding diagnosis and treatment is not fully accounted for by appeal to individual knowers and does not do justice to the complex coordination of team members with different expertise, where social interactions play a pivotal role. The social epistemology we propose is better able to develop an understanding of diagnosis and management decisions made in clinical teams that are becoming increasingly complex. Rapid changes in the availability and quality of imaging, the development of new and expensive drugs, and an increasing realisation of the need to place medicine in a social context for patient benefit have driven the multidisciplinary team (MDT) to come to the fore as a central place for shared decision‐making.

The essential role of technologies is also not normally included in accounts of knowledge in clinical decision‐making. Technologies are normally relegated to vehicles for evidence, but we argue that technologies, and evidence and knowledge claims made on their basis, coevolve with each other and play an essential role in mediating the social knowledge of clinical contexts. In this regard, images are most often analyzed in terms of their ability to provide evidence for (scientific) claims. For example, Perini^23^ analyzes how mechanically produced images are structurally related to the shape of the specimen being imaged by being sensitive to certain aspects of a specimen and indifferent to others. However, most medical imaging techniques are not purely mechanically produced, but have a substantive informational component too. For example, Carusi^24^ argues “embodied in the algorithm for image processing, there is a hybridity of causal factors (the way in which the algorithm organises shapes and contours in the image) and intentional/ informational factors. The resultant images that are viewed for further interpretations are a hybrid of causal and non‐causal factors.” In other words, the image is not the result of a chain of causal factors, but of causal factors combined with factors like processing algorithms, that are programmed with an intention to filter, simplify, or interpolate data. Hence, medical images cannot be simply regarded as “vehicles for seeing‐in” the body and image technologies as “visual prosthetics” that provides direct access to the inside of patients.^25^

In addition, several authors have argued that the process of image analysis by radiologists or clinicians can be understood as a *hermeneutic system*. According to Friis^26^ the interpretation of images takes place preconsciously. He characterises image interpretation in terms of the hermeneutic circle, in which the mind moves from parts to whole and back to make sense of an image. Friis invokes the concept of *gestalt* to analyze the interpretation of medical images. In his words, gestalts are “something that stand out against a background and enables us to identify patterns.” and that cannot be understood as a feature of the image itself, but of the image in interaction with the perceiver. The perceiver is in turn shaped by his or her background: Friis argues that visual perception is an embodied skill that is shaped by biology, society, experiences and training that together make up a personal “horizon”, meaning that visual perception is variable from person to person. Therefore, 2 radiologists may interpret the same image differently, because of variability in their horizon. Rosenberger^27^ argues that most images are multistable, meaning that they can be interpreted differently by applying a different hermeneutic strategy, informed by “theoretical commitments, explanations of the structures within the image's content, and relations to the imaging technologies.” He applies this theory to an ongoing debate in neurology, where the 2 opposing parties interpret the same images differently. Questioning each party's hermeneutic strategy, Rosenberger argues, can suggest further trajectories for research by asking for a more detailed account of the morphologies present in the image according to rivalling theories. These authors give an account of how image and interpreter interact, but omit an understanding of how this interaction shapes the social distribution of knowing and mediates the interaction between different members of clinical groupings. This is what we focus on in this paper. In her article on computed tomography (CT) images as diagnostic tools, Friedrich^28^ proposes that the interpretation of these images depends upon the development of shared “sight styles” across radiologists in a clinic. Her account draws upon Ludwik Fleck's notion of “thought styles” and “thought collectives,” stressing the social processes and the role of technologies such as software, through which people come to see in the same way.^29^, ^30^ As will become apparent, the account we propose here is similarly oriented toward the development of shared modes of seeing.

In summary, we will argue that rather than focusing on the individual clinician's reasoning and knowledge, it is more fruitful to think of clinical decision‐making as a form of social knowing, in which technologies play a key mediating role. In such a system, decision‐making cannot be performed by any one individual, but is instead performed by an assemblage of people and instruments in coordinated actions. This paper examines clinical decision‐making through a detailed study of image‐assisted diagnosis and treatment of a pulmonary disease. The study shows the knowledge processes involved among the different epistemic agents with different expertise who collaborate on formulating decisions. We will show how in repeated interactions, medical teams cultivate a collection of stable, agreed upon orientations toward evidence and knowledge that establishes an intersubjective framework within which claims and interpretations can be justified and decisions can be arrived at and shared by others. Medical images, such as X‐rays and magnetic resonance imaging (MRI) scans, play an important role in these assemblages of distributed knowing.

## 2 METHODS

We have analyzed clinical decision‐making within a medical expert team involved in diagnosis and treatment of patients with a complex disease called pulmonary hypertension (PH), over the course of 9 weeks. PH is a rare and life‐shortening disease characterised by an elevation of blood pressure in the pulmonary artery and an increased resistance in the pulmonary vasculature.^31^ This results in an enlargement and decreased function of the right heart ventricle that causes breathlessness and limitation of exercise capacity that may be very severe.^32^ PH has a definition given in terms of a measure produced by an invasive test of right heart catheterisation. It is further classified into 5 different categories (with a number of subdivisions) according to cause, 1(1) Pulmonary arterial hypertension either idiopathic or associated with other conditions, (2) pulmonary hypertension (PH) due to left heart disease, (3) PH due to lung diseases and/or hypoxia, (4) chronic thromboembolic PH, and (5) PH with unclear and/or multifactorial mechanisms. for which different treatments are required.^33^ Images play a crucial role in establishing the cause of particular cases of PH and are therefore important diagnostic tools. A careful clinical history and a range of investigations are required to diagnose and categorise PH. The treatment regime for the patient is based upon these tests and classifications and can range from drug treatments to heart and/or lung transplantation.

The medical team in our research worked in 1 of 8 expert centres in the United Kingdom and Ireland that diagnose and treat PH and consists principally of pulmonary clinicians, a cardiologist, a nurse consultant, specialist pharmacists, radiologists, junior doctors, specialist nurses, and a ward nursing team. Although a study of clinical decision‐making is not complete without patients, patients were not included in this particular study for pragmatic reasons alone, as our ethical clearance did not extend to them. For this reason, the study focuses on how images are used for diagnosis, which is an aspect of the decision‐making process where the division of epistemic labor falls more on the clinical team; a fuller study will also consider patients.^34^ Participants in the study were all members of the clinical team and invited to contribute to the study and/or collaborate on it. The team confers weekly in a ward MDT meeting discussing the management of the current ward patients and a radiology MDT meeting where current inpatients, patients in short‐stay admissions for diagnostic testing, and outpatients may be discussed. Data were collected through observing weekly MDT meetings, performing 11 qualitative semi‐structured interviews with members of the clinical team, and conducting a group discussion on emerging imaging technologies. MDT meetings were not video or audio recorded as we did not have ethical clearance for this; we recorded our observations in notes. In addition, we video‐recorded a session of 2 radiologists collaboratively reporting an X‐ray CT scan and an interdisciplinary meeting to determine the usefulness of an emerging imaging technique. All recordings were transcribed and coded using nvivo (QRS international Pty Ltd. version 10, 2012). The data used for this particular study are the semi‐structured interviews, which had framed our data collection in terms of expertise, teamwork, and the role of imaging technologies. The interviews were divided into 3 main sections: the interpretation and use of images, expertise and trust, and the introduction of new imaging modalities. The analysis of the data broadly followed these categorisations, but also looked for connections between them, using a grounded approach, that is, using the main topics and subtopics of the interviews as a first iteration, and an open coding approach, looking for relationships and groupings within and among these topics and subtopics, thereby establishing recurring and contrasting motifs and themes. In particular, we looked for connections between the MDT observations and the interviews with individual research participants, as is evidenced in the discussion below.

## 3 RESULTS

### 3.1 Social knowing in clinical decision‐making

Decision‐making in the PH clinic involves combining evidence from heterogeneous sources, such as the patient's history, clinical examination, lab tests, images and measurements, awareness of personal and social circumstances, observations of the patient by clinicians on ward rounds and by ward staff, or interactions with family. One of the key epistemological challenges of clinicians is to develop an account of every individual patient based on the available evidence, a process that involves the interpretation and adjustment of pieces of evidence so that they form a cohesive and consistent “picture” 2We use the term ‘picture’ as it is used colloquially in the domain of our fieldwork, without any commitment to representationalism. A non‐representational account of pictures is not at all unusual; see footnote 7. of that patient.^35^ These pieces of evidence are generated and interpreted by different people; for example, radiographers generate images by operating the imaging apparatus when the patient is scanned, and by doing initial data processing—which is in a sense, already a first form of interpretation; radiologists interpret the images, but so too do clinicians, with different levels of expertise. Nurses and clinicians generate evidence through the patient history and clinical relationship, and their interpretation provides the clinical questions that radiologists use to direct their interpretation of the images. In these respects, the process is distributed over people, working in different and overlapping contexts, at different points of the patient's encounter with the clinic. Part of the adjustment and interpretation of evidence occurs within interactions with different experts, who from their different expertise provide a specific outlook on evidence while fitting this in with other evidence requires the interpretations of other experts. MDT meetings play an important role in socially distributed knowing, for instance, by providing a space where information and interpretations can converge into a shared team decision, as also described by Maseide^14^ and Cicourel^13^.

In our epistemological analysis of team decision‐making in the PH service, we will demonstrate how MDTs need to combine the individual expertise of team members to be able to fit together all relevant information that leads to a team decision and that therefore knowing in distributed.

### 3.2 MDT meetings

**Figure 1:**
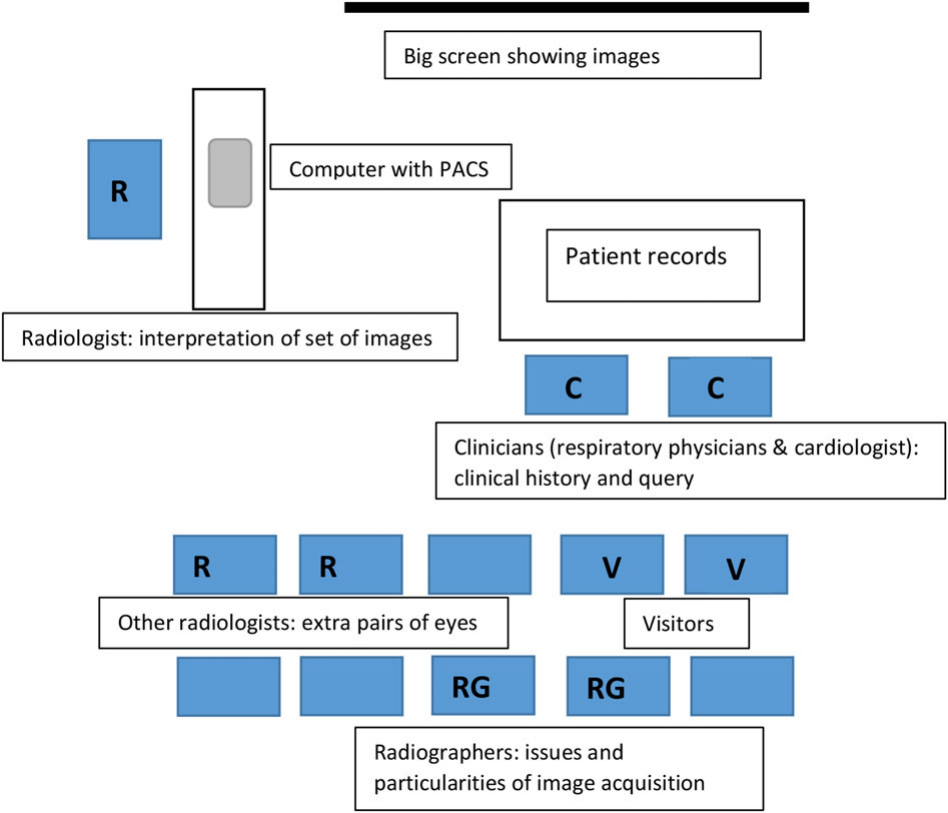
Room layout and contributions at weekly radiology multidisciplinary team. PACS, patient archiving and communication system

We observed 2 types of MDT meetings: on the ward and in the radiology section. Here, we focus on the second of these. In weekly radiology meetings, the PH team of our field study reviews the imaging of all patients in the last week, admitted for diagnostic testing, or admitted for acute management of a deteriorating clinical condition, and patients who are being (re)evaluated as outpatients. See Figure 1 for an overview of the seating in radiologist MDTs. Radiologists have prepared the meeting by reporting the available images and take a place behind the workstation to navigate the different images using a patient archiving and communication system (PACS). Images are shown at a large screen in front of the room, while clinicians (consultant respiratory physicians and a consultant cardiologist) take place at a table at the front of the room, consulting the medical records of the patients. Other attendees at MDTs are the radiographers who have scanned last week's patients, junior doctors, registrars, and visitors. At most meetings at least 2 radiologists and 2 consultant clinicians are present. A usual interaction concerning imaging of a patient is structured as follows: the clinician opens with an introduction of the patient, a summary of the previous course of the disease, clinical signs and symptoms, other test results, and sometimes a specific question. The radiologist then draws up the images, compares results from different imaging modalities, and compares current images with earlier images if available. They show specific findings in the images and sometimes ask for clarification about the patient's clinical history from the clinician to refine their evaluation. After a series of interactions, the radiologist summarises his or her view, with a response to any the initial question, after which the clinician concludes the interaction by making a note of the shared conclusion and the follow‐up plan for that patient, which they say out loud while writing it down in the patient's clinical record.

In these interactions, heterogeneous sources of knowledge and information are fitted into a group decision by hearing voices that represent a range of expertise. Team members have different knowledge and skills that stem from different backgrounds in training, but different experts also interact differently with individual patients, their bodies, and medical instrumentation such as imaging. For example, the clinician has met the patient, has taken his or her medical history, performed a medical exam (eg, listened to their heartbeat using a stethoscope), and has studied lab results. Radiologists receive only a short summary of the patient's history and the clinician's query, but spend much time studying and interpreting the imaging results. Clinicians decide whether a patient will go for imaging or not, which imaging modalities are used and which questions are asked, based on their knowledge of the patient and in the context of a diagnostic and management process aimed at understanding and treating an individual to improve symptoms and prolong life. Radiologists are guided by these questions, without having full access to how the clinician came to that question or what were their reasons for requesting a certain imaging exam for this patient. Conversely, physicians are guided by radiologists' interpretation of their patient's images to make clinical decisions, without having full access to their interpretation, or the expertise that leads them to make the interpretation (see Quote 1).

Quote 1: *Obviously, you can*'*t read the entire patient notes. So you want a clear summary of what the patient has. Brief. And the best thing is if you*'*ve got a clear question of what you want to ask from the imaging. Sometimes the imaging* *…. you could come to multiple conclusions, but if you*'*ve got a clear question that makes it a lot easier because you can answer that question and then everything else can be kind of incidental finding, if you find other things. (P3, radiologist)*

In short, within these groups, the epistemic labor is distributed over different specialisms and experts, each with different roles and epistemic contributions. For example, radiologists are experts in the way anatomical or pathological structures appear on different imaging modalities, whereas clinicians have a complex understanding of pathological processes and how these present as signs and symptoms in patients. In exchanges in MDT meetings, these different expert approaches help interpret the separate pieces of evidence in relation to other available evidence, providing a refinement and enrichment of these interpretations that a single expert would not be able to reach on their own (eg, see Quote 2). Even though there are different roles and expertise, they must overlap sufficiently for the interactions among members to be meaningful; for example, clinicians in this group described themselves as having more expertise on the images relating to PH than other radiologists who do not specialise in this area. What is generated is a palimpsest of overlapping and superimposed knowledge rather than a jigsaw made up of discrete pieces that fit together.

Quote 2: *And that's why an MDT environment is so important* *... because* *... you need to have those cautionary people who understand the limitations, so usually the radiologists, to be able to advise the clinical team, about your level of confidence. (P8, radiologist)*

These refined and enriched interpretations help to bring heterogeneous information together, into a shared framing of the patient, a collective understanding of the patient's illness that is built up from all pieces of evidence, where exchange between different expertise is necessary to be able to adjust and reinterpret the available evidence to be able to make them fit. Building this shared “picture” of the patient allows them to come to a shared conclusion concerning the diagnosis and treatment for him or her, which is usually written down in the patient's clinical record while being voiced out loud by the consultant clinician. This voicing out loud underscores the shared ownership of the team's conclusion and decision (see Quote 3).

Quote 3: *Actually within our services it*'*s rather more about you take the opinions of your colleagues and the knowledge that you have as a team, and you work out how to apply that with yourself as an instrument of the team. So you*'*re not placing yourself above anything, but it*'*s more that you*'*re filtering, taking in all things that you*'*re told and trying to work out the best fit. (P1, consultant respiratory physician)*

MDT meetings are geared toward consensus within the full team. In most meetings, more than one radiologist and more than one clinician are present, to have an extra pair of eyes and make sure that things are not missed, and also because a conclusion or interpretation that is shared by others is considered more reliable than when it is reached by a single person. Hence, one of the purposes of MDT meetings is to deal with uncertainty and incomplete information, which is a challenge of clinical decision‐making.^3^ The consensual nature of this process is one way of managing responsibility for the patient, and we would expect that distributed knowledge also leads to distributed responsibility.

Quote 4: *That's why it's really good we have a group of at least 2 or 3 radiologists in the team. So we take consensus from the others as well, from the imaging. And if they all agree, than it's more reassuring and then we can say on our report or in the discussion, so for imaging this is what it is (P4, radiologist)*

Members of the unit frequently mentioned that working in a team gave them more confidence in their decisions, see, for example, Quotes 4 and 5. 3The consultant clinician is ultimately responsible for the patient, legally and in the organisational structure of the hospital; however, there are tensions between this ultimate legal and organisational responsibility and the consensual nature of the decision‐making process. These are interesting and important issues, but space does not allow them to be explored here.

Quote 5: *Usually people come to a consensus. Ehm* *... and* *.... it's not just the two radiologists either, because there's a lot of expertise in the imaging from the clinicians as well. So we have like a collective* *... opinion.”* (P3, radiologist)

Hence, the process of fitting together heterogeneous sources of information into a coherent and consistent framing of a patient is a collaborative effort, and MDT meetings are an important place where information can be shared, discussed, interpreted, and adjusted, allowing the development of a collective way of seeing and a shared language. For example, after voicing the team's decision, the consultant clinician also mentions the right heart catheter measurements, which allows for a final check and last integration of all evidence and which helps radiologists to get a feel for the correlation between the imaging and right heart catheter findings. MDTs continue to play their role when clinicians or radiologists are not in a meeting, first by knowing that they are held accountable for the quality, relevance, and comprehensiveness of the information they provide at MDT meetings and second by shaping the information that is gathered in such a way that it fits the structure of the shared framework of the MDT.

In terms of *epistemological responsibilities*, it is interesting that these include responsibilities toward the sociability of the team. Team members have an epistemological responsibility to weigh up evidence according to their knowledge and also to open up their deliberation to others, justifying to others how they come to a certain interpretation while being sensitive to deliberations and interpretations from others. If we take an overarching epistemological responsibility of each person to be toward a sound shared decision regarding the care of patients, an aspect of that *epistemological* responsibility is inherently *social* in character. This means that it is not an epistemological plus a social responsibility, side by side, *but both at the same time.* This is seen in the more detailed example of image interpretation in the next section.

### 3.3 Distributed knowing in image interpretation

The structure of distributed knowing is especially clear when dealing with images. Kelly Joyce (2005) demonstrated that the use of MRI is local, embodied and contingent, for three reasons.^36^ Firstly because in the production of the image parameter choices by radiographers while physically scanning a patient influence the resulting image, secondly because the interpretation and translation of the image by trained radiologists produces a report that remains open to divergent interpretations and lastly because imaging can conflict with other available information and can be taken up by the clinician in various ways. No one in the PH team in our field work has complete knowledge of the images. Although radiologists are considered the experts when it comes to medical images, they perform their role within an assemblage of other medical professionals and instruments. As argued above, to fully evaluate imaging, it is necessary to fit with other evidence and interpretations of this evidence by other experts, such as the clinical story as provided by the clinician. In addition, important components in this knowledge‐generating assemblage are the physical MRI, the work of the radiographers who operate the scanner and instruct the patient and subsequently process the data to produce high‐quality images and metrics of the right kind, and software systems such as PACS to share, view, and analyze imaging and to add reports. None of these components, clinicians, radiologists, radiographers, scanners and software, can be omitted from an account of knowledge generation in the context of PH diagnosis.

For example, the PH team makes use of an MRI scan called cardiac magnetic resonance (CMRI) to assess the anatomy and function of the cardiac chambers. A typical CMRI sequence requires synchronisation of MRI information with a person's heart rhythm as measured via electrocardiogram for as long as 40 minutes, which enables reconstructing a moving image of the heart during the cardiac cycle (this process is called “cardiac gating”). This image sequence resembles a beating heart, and this is used to assess the function of the heart by visual assessment of chamber anatomy, contraction, and potential leaking of heart valves. The right heart function is relevant for prognosis and disease severity, while the left heart is assessed to exclude left heart disease. In addition, images are processed to quantify predetermined parameters relevant to cardiac function, such as ejection fraction (the amount of blood pumped by the heart with each heartbeat) and calculated cardiac output (the amount of blood pumped per amount of time). For example, to measure the right ventricular ejection fraction, a measure held to be clinically important by correlating with disease severity and prognosis, the volume of the right ventricle is measured at two moments in the cardiac cycle: immediately before contraction (the end‐diastolic volume) and immediately after contraction (the end‐systolic volume). This is done by drawing the contour of the right ventricle in all slices covering the right ventricle volume for the two points in the cardiac cycle.^37^ A radiographer draws the right ventricle contours after which a software program calculates the ejection fraction and other metrics characterising the right ventricle function that are summarised in a report containing numbers and diagrams that the radiologists receive in PACS.

The above description of the production of one MR metric employed to make clinical decisions demonstrates how knowing in clinical practice is socially distributed. Radiographers make a knowledge claim by drawing the contour of the right ventricle, defining which part of the image refers to the ventricle wall and which to the inside of the ventricle. They are able to make such a knowledge claim because they have developed a way of looking at these images, as part of their education and experience, but more importantly in interaction with other experts, such as radiologists and researchers, who establish the relevance of this knowledge claim—demarcating the border of ventricle wall—by relating it to a clinically relevant metric—the right heart ejection fraction. For radiologists to evaluate the right ventricle ejection fraction, and thus make a knowledge claim about disease severity or prognosis, they require knowledge claims made by radiographers regarding the right ventricle wall in order for the measure to be processed. The two types of knowledge claims develop in tandem with each other, through iterative cycles during which the border of the right heart ventricle is picked out for clinical relevance, and the radiographers draw the border in such a way that the radiologists can use it for their decisions. In short, knowledge in this small instance is distributed over at least two people who both play a crucial role in generating an evaluation of the right heart function, and who both play their roles embedded within a broader team and other interactions, with other radiographers, radiologists, and consultant clinicians. No one person has complete knowledge of the images, and the knowledge each does have is in virtue of their interactions with others. Each person is responsible for how he or she contributes to the knowledge of others and not only to their own piece of the puzzle. This contribution includes responsibilities toward openness to others and recognition of oneself as part of a team.

### 3.4 The mediating role of imaging technologies

In our field study, the physicists, radiographers, and radiologists involved in PH imaging are highly specialised and have a long history of collaboration, developing methods to analyse and evaluate CMR images and metrics together. The technologies, for example, the scanner, the sequences facilitating the acquisition of CMR images, the image processing algorithms and the software tools that enable drawing the right heart ventricle contours, calculating the ejection fraction and sharing the results, play a crucial and active role in these processes. The technologies, the users, the ways of looking, and the possible knowledge claims coevolve with each other. Kelly Joyce (2006) demonstrates how MRI coevolved with ways of looking by describing its historical development.^38^ MRI was originally developed as a tool to measure the composition of materials in physics and chemistry (spectroscopy) and later, driven by the “war on cancer” in the United States, attempted to be modified into a tool to measure tissue composition and ultimately into an imaging method. The images produced by MRI were initially in full color and included an array of numbers. After being taken up by radiology, MRI scans were presented in gray scale, fitting the images radiologists were already familiar with and the existing technological constraints. In the field of PH, this coevolution is also evident. MRI, by producing a specific type or contrast, between different types of soft tissues, drives a specific kind of visualisation of the heart muscle, and the method of electrocardiogram‐gating allows visualisation of movements of the heart during a complete heart cycle, enabling CMRI. Clinicians and radiologists involved in diagnosis and treatment of PH, from being familiar with heart anatomy and physiology, recognise the relevant structures (ie, septum, ventricles, and valves), and from being familiar with what type of information is required in clinical practice, they recognise which relevant questions might possibly be answered by these types of imaging. However, they need to learn how to recognise deficiencies and how to evaluate function by relating images to clinical outcomes. Together with an ongoing and rigorous discussion, these interactions among radiographers, radiologists and clinicians, and the imaging technologies, push the development and tweaking of acquisition sequences to improve image contrast for those specific practices, and image processing and analysis algorithms to produce relevant metrics such as right heart ventricle ejection fraction.

In repeated interactions, medical teams cultivate a collection of stable, agreed upon orientations toward evidence and knowledge that provides an intersubjective framework within which claims and interpretations can be justified and decisions can be arrived at and shared by others. Medical images play an important role in the building of these intersubjective frameworks in three ways: first, as enablers or tools in acquiring information; second, as communication facilitators; and third, as pervasively framing the epistemic domain.^24^, ^39^, ^40^ Through these 3 mediating roles cumulatively and simultaneously, imaging modalities are active shapers of the epistemic domain, for example, by shaping what counts as evidence for specific diagnoses and by shaping classificatory structures and treatment régimes for diagnoses.

That images are enablers or tools in acquiring information is clearly evident throughout the history of imaging, as from the first X‐ray, images have been a powerful means of pushing back the limits of observation. In the PH field, continued research on imaging such as CT and MRI has allowed a visual detection of several mechanisms causing PH (eg, chronic blood clots in the lung, lung emphysema, left heart disease, etc) leading to more reliable clinical categorisation and development of specific diagnosis and treatment approaches according to PH‐group. Because images play such a crucial role in the diagnosis and management of the disease, they play a prominent role in interactions and communications among the members of the medical team. 4Images might also play a role in the interactions between clinicians and patients; however, we did not study these interactions in our field work. This is obvious in the radiology MDTs, where the images are discussed, but also clear in the ward MDT meetings. These are led by different consultant clinicians in different weeks, and there is variation in the display and reference to displayed images depending on which consultant clinician leads; mostly the images relating to the patient discussed are displayed and discussed; at the very least, they are always mentioned and referred to. 5An imaging modality that plays an important role in PH diagnosis is the echocardiogram, but interestingly, this modality is hardly ever referred to, let alone displayed at multidisciplinary team meeting. This probably has to do with the fact that echocardiograms are not shared via the same patient archiving and communication system, with the user‐specificity of US images and that interpretation of these images requires cardiologic expertise. Another modality that is relatively little referred to is electrocardiography.


The third mediating role of images, as pervasively framing the epistemic domain, is closely related to its other two roles, but relates to the sheer scale of image use and research in the domain. MRI became routinely used in the PH unit that we studied since 2004. 6The development of magnetic resonance imaging methods for PH diagnosis and classification and the impact of these are another very interesting topic, but go beyond the scope of this paper and will be discussed in other papers. Since its introduction, even artifacts of CMR imaging have been found to be diagnostically useful, as in the case of the so‐called black blood artifact,^41^ and other imaging technologies and other tests become less used. The introduction and development of a new imaging technique or modality usually runs alongside and piggybacks on an existing one, as it needs both to cohere with and go beyond the existing techniques and modalities. As we have discussed, the ability to interpret and use the images coevolves with the development and implementation of the technology, and the expertise and skill with which images are interpreted are built up through continued use over lengthy periods of time, in interaction with other people. When an imaging modality overcomes a critical point and becomes dominant, it pushes out other preexisting modalities. For example, in Quote 6 there is a radiologist describing how MRI perfusion in combination with accurate CT pulmonary angiography came to be used in their unit as an accurate, noninvasive test that ultimately replaced the more invasive pulmonary angiogram:

Quote 6: *P4…so looking at perfusion you could see the lungs taking up contrast. So if it*'*s even in both sides of the lungs, then that's fine. But if you see like defects, you know like big chunks out of contrast that's missing. Than that's a feature of chronic thromboembolic disease. So we started noticing those.. changes.*

*[…]*

*P4: before, we used to use, just* ***contrast angiography*** *, so what we used to was, to look at the pulmonary arteries, we used to just inject contrast and then just look at the flow of the contrast in the pulmonary artery. So very* *... it's an* ***invasive procedure*** *, so you have to have a catheter put in the groin and* *... but, that's more or less obsolete these days. So we don't, we hardly do* *one a year.*

*Interviewer: do you notice anything* *... do you miss anything about images that* *…. modalities that you, you know* *... is there any time that you would say, well we could have seen that on* *...[a pre‐existing modality]*

*P4: the thing is, because we don't do it that often, we're losing the skill to interpret the* *...*

*[…]*

*P4: So, you know, if somebody gives us a pulmonary angiography now, I think we'll all struggle to identify what's happening.*

When imaging modalities became so embedded into the epistemic domain, it becomes difficult to get an external vantage point on them, and they, in their turn, become the standards against which continued imaging developments are assessed. The evidence for defects in the lung, for example, comes to be constituted by how this is visualised in perfusion MRI. In this way, images are pervasive mediators that can reshape the epistemic domain.

Thus, the epistemology of clinical decision‐making is also ineliminably technological as well as being social; in fact, these two aspects cannot be divorced from each other, as they are one in virtue of being the other. The most powerful technological means for probing a clinical domain cannot be used, cannot even have meaning for that domain, outside of the social relations through which interpretations are engendered and decisions are grounded. However, this very process of using technologies effectively—which, we have seen, requires expertise and skill that are honed through social interactions—also makes it difficult to arrive at purely external assessments of specific technological developments once they become the norm, because the process of producing the expertise to interpret them can also, paradoxically, remove the ground for making a comparison.

## 4 DISCUSSION

The study we conducted is a small qualitative study of clinical decision‐making in diagnosis and treatment of PH, involving only one team in a relatively short frame of time. In this study, we focused on images and the clinical team using and developing them, whereas further studies need to broaden this out to consider others in the process, in particular nurses and patients; in addition further comparative studies of other PH teams would enable us to discover how specific our findings are to this team. Even so, we believe that it points to some important features of the social epistemology of image‐mediated clinical decision‐making. We have argued that clinical decision‐making is highly social and mediated by technologies, in this case imaging technologies. Imaging, the ability to interpret images, social practices, and the epistemic domain codevelop into a socio‐technical epistemic framework in which members of the clinical team exchange, discuss, and fit together evidence toward a team opinion. These aspects of clinical decision‐making mean that an individualist epistemology is inadequate. Instead, the epistemology of clinical decision‐making is ineliminably social and technologically mediated.

In this article we have emphasised the social nature of knowledge in the process of coming to a shared way of seeing, or what Friedrich^28^ labelled “sight style.” This differentiates our account of socio‐technological epistemology from the tradition of distributed cognition, as we do not invoke internal or external representations. Of course, the word “representation” is frequently used in the clinic as elsewhere in scientific contexts, but our emphasis has been on how something comes to be agreed upon as a representation, and we do not take for granted in advance that anything actually is a representation because this assumes that it is already or *a priori* clear how to interpret it as a representation. On our account, it is the process whereby an image's status as being a representation of some aspect of the clinical situation is established, that is at issue: as in our example, images come to represent the size of the right heart ventricle through an interactional interplay between radiologists and radiographers that foregrounds the border of this ventricle and establishes a way of drawing it, rather than this being pre‐given. 7There are further deep differences between our view and distributed cognition that cannot be dealt with here. These are briefly differences on the status of internal representations (our account takes a phenomenological approach and bypasses these entirely); the espousal of many cognitivist accounts of a computational theory or metaphoric framework of cognition, which we do not adopt; and the view that (for example in Cunningham 2014: 187) artifacts are a subset of tools that assist people to perform cognitive functions that they could otherwise perform for themselves.^22^ In the same tradition, images and visualizations have also been understood as distributed representations that aid visual thinking and communication in distributed cognitive systems.^42^ On our account, artifacts, images, and visualizations are not only aids to thinking and communication, as this would take into account only the first and second mediating roles described above; rather, they have a further active mediating role in establishing a shared way of seeing as a first step to shared modes of thought. The view taken in this article is an extension of nonrepresentationalist accounts of images and models that 1 of the authors has been systematically developing in several publications, for example, Carusi (2016), Carusi and Hoel (2015) (see also further references to Carusi & Hoel publications on this topic in those articles), and Carusi (2012).^24^, ^43^, ^44^ Nonrepresentationalism about perception and knowledge is not a new position, but was most significantly advanced in philosophy by the phenomenology of perception of Maurice Merleau‐Ponty (1962; originally published in 1945)^45^; since then it has had numerous proponents, including significant elaborations of the position in social sciences by, for example, Lynch (1988), Goodwin (1994), Goodwin (1997), Sharrock and Coulter (1998).^46^, ^47^, ^48^, ^49^ In the tradition of cognitive sciences, it is espoused most notably by Noë (2004).^50^



Furthermore, our analysis implies that it is not enough to focus on the epistemological responsibilities of knowers operating as individuals, but that to be able to understand the domain better, we also need to understand how epistemological responsibilities include responsibilities toward sociability and technological mediation. In other words, epistemological responsibilities of physicians not only include the gathering, interpretation, and fitting together of evidence for each patient but also include an openness toward evidence and interpretations, and knowledge claims made on their basis by other team members, and making one's own interpretations accessible to others.

The socio‐technological epistemology that we are proposing opens up several questions for further investigation: we have pointed to issues about responsibilities of clinical decision‐making that need further analysis, as well as issues in the development and validation of new technologies and imaging tools. We end on a note regarding the potential of the socio‐technological epistemology we propose to open up new roles for philosophers and social scientists in participating in the formation of clinical teams. Rephrasing John Hardwig quoted earlier in the paper, our epistemological analysis of the social structure of clinical decision‐making suggests that attending to the sociability of clinical decision‐making is an essential aspect of what “makes the members of some teams knowers while the members of others are not”. This attention is something that philosophers and social scientists could contribute to the understanding of clinical decision‐making.
